# Biallelic loss of *EMC10* leads to mild to severe intellectual disability

**DOI:** 10.1002/acn3.51602

**Published:** 2022-06-09

**Authors:** Rauan Kaiyrzhanov, Clarissa Rocca, Mohnish Suri, Sughra Gulieva, Maha S. Zaki, Noa Z. Henig, Karine Siquier, Ulviyya Guliyeva, Samir M. Mounir, Daphna Marom, Aynur Allahverdiyeva, Hisham Megahed, Hans van Bokhoven, Vincent Cantagrel, Aboulfazl Rad, Alemeh Pourkeramti, Boshra Dehghani, Diane D. Shao, Keren Markus‐Bustani, Efrat Sofrin‐Drucker, Naama Orenstein, Kamran Salayev, Filippo Arrigoni, Henry Houlden, Reza Maroofian

**Affiliations:** ^1^ Department of Neuromuscular Disorders Queen Square Institute of Neurology, University College London London UK; ^2^ Clinical Genetics Service Nottingham University Hospitals NHS Trust Nottingham UK; ^3^ MediClub Hospital 45, Uzeyir Hajibeyli str. Baku AZ1010 Azerbaijan; ^4^ Human Genetics and Genome Research Division, Clinical Genetics Department National Research Centre Cairo Egypt; ^5^ Genetics Institute Tel Aviv Sourasky Medical Center Tel Aviv Israel; ^6^ Developmental Brain Disorders Laboratory, Imagine Institute, INSERM UMR Université Paris Cité Paris France; ^7^ Pediatrics Department, Faculty of Medicine El‐Minia University Minia Egypt; ^8^ Sackler Faculty of Medicine Tel Aviv University Tel Aviv Israel; ^9^ Child Neurology Hospital Taghi Shahbazi str. Baku AZ1065 Azerbaijan; ^10^ Clinical Genetics Department National Research Centre Cairo Egypt; ^11^ Deparment of Human Genetics, Donders Center for Brain Cognition and Behaviour Radboud University Medical Center Nijmegen the Netherlands; ^12^ Department of Otolaryngology, Head and Neck Surgery, Tübingen Hearing Research Centre Eberhard Karls University Tübingen 72076 Germany; ^13^ Medical Biotechnology Research Center Ashkezar University Ashkezar Yazd Iran; ^14^ Department of Neurology Boston Children's Hospital Boston Massachusetts USA; ^15^ Division of Genetics and Genomics, Department of Pediatrics Boston Children's Hospital Boston Massachusetts USA; ^16^ Raphael Recanati Genetic Institute, Rabin Medical Center‐Beilinson Hospital Petach Tikva Israel; ^17^ Department of Pediatric Genetics Schneider Children Medical Center of Israel Petah Tikva Israel; ^18^ Department of Neurology Azerbaijan Medical University Baku Azerbaijan; ^19^ Paediatric Radiology and Neuroradiology Department V. Buzzi Children's Hospital Milan Italy

## Abstract

The endoplasmic reticulum membrane protein complex subunit 10 (EMC10) is a highly conserved protein responsible for the post‐translational insertion of tail‐anchored membrane proteins into the endoplasmic reticulum in a defined topology. Two biallelic variants in *EMC10* have previously been associated with a neurodevelopmental disorder. Utilizing exome sequencing and international data sharing we have identified 10 affected individuals from six independent families with five new biallelic loss‐of‐function and one previously reported recurrent *EMC10* variants. This report expands the molecular and clinical spectrum of *EMC10* deficiency, provides a comprehensive dysmorphological assessment and highlights an overlap between the clinical features of *EMC10*‐and *EMC1*‐related disease.

## Introduction

The endoplasmic reticulum membrane protein complex (EMC) is a highly conserved, multifunctional, and multi‐subunit protein complex responsible for the post‐translational insertion of tail‐anchored membrane proteins into the endoplasmic reticulum (ER) in a defined topology.^1^ The insertion of proteins into membranes is an essential cellular process influencing vesicular trafficking, apoptosis, signal transduction, and lipid biosynthesis.^2^ The mammalian EMC is composed of 10 subunits, EMC1–10,^3^ encoded by the corresponding EMC1 − 10 genes, two of which have currently been linked to human disease. Thus, biallelic variants in *EMC1* have been linked to the cerebellar atrophy, visual impairment, and psychomotor retardation syndrome (MIM: 616875),^4^, ^5^, ^6^, ^7^ and hitherto two homozygous loss‐of‐function (LOF) variants in *EMC10* have been associated with the neurodevelopmental disorder (NDD) with dysmorphic facies and variable seizures (MIM: 619264) in eight families.^8^, ^9^, ^10^ The two previously reported disease‐causing *EMC10* variants include a homozygous splice acceptor site variant c.679‐1G>A found in two siblings from a Saudi Arabian family and a recurrent homozygous frameshift variant c.287delG, p.Gly96AlafsTer9 identified in 13 affected individuals from seven families of Bedouin, Saudi Arabia, and the United Arab Emirates origin.^8^, ^9^, ^10^

Herein, we present 10 affected individuals from six unrelated families harboring five previously unreported and one recurrent homozygous LOF variants in *EMC10*. The report expands the clinical spectrum of *EMC10*‐related NDD towards the more severe end, highlights an overlap between the clinical features of *EMC10*‐and *EMC1*‐related disease, and discovers new disease‐associated *EMC10* LOF variants.

## Methods

### Participants**'** recruitment, data collection, and clinical and genetic investigation

By exome sequencing (ES) of families affected by undiagnosed NDD, data mining of DNA sequences from families with rare disorders aggregated across several diagnostic and research laboratories, and using GeneMatcher data sharing platform^11^ six independent families reported here were identified. A uniform clinical proforma was distributed to collect clinico‐demographic details. Facial photographs and brain magnetic resonance imaging (MRI) scans were obtained from the present cases and were retrieved from the previous reports^8^, ^9^ for a review conducted by an experienced dysmorphologist and pediatric neuroradiologist (M. S. and F. A.). Parents and legal guardians of all affected individuals gave their consent for the publication of clinical and genetic information according to the Declaration of Helsinki, and the study was approved by the respective local Ethics Committees. Follow‐up details on the parathyroid gland function and levels of global developmental delay/intellectual disability (GDD/ID) were obtained from the cases reported in Shao et al.^9^ Trio ES or solo ES with subsequent Sanger segregation analysis was carried out in DNA extracted from blood‐derived leukocytes in six families in four different centers following slightly different protocols (Table 1 for methods). ES data analysis, variant filtering, and prioritization were performed using in‐house implemented pipelines of the local genetic centers (Table 1 for methods). In brief, the bioinformatics filtering strategy included screening for only exonic and donor/acceptor splicing variants. In accordance with the pedigree and phenotype, priority was given to rare variants (<0.01% in public databases) that were fitting a recessive (homozygous or compound heterozygous) or a de novo model and/or variants in genes previously linked to NDD/ID and other neurological disorders.

**Table 1:** Clinical features of the *EMC10* cohort, genetic methods, and variant characteristics.

		Patient	Family 1	Family 2		Family 3			Family 4	Family 5	Family 6		Umair et al. (2020)	Shao et al. (2021)
			S1	S2	S3	S4	S5	S6	S7	S8	S9	S10	2 cases 1 family	13 cases from 7 families
Variant annotation		Variant type	Frameshift	Frameshift		Stop gained			Stop gained	Frameshift	Splicing	Splicing	Splice acceptor	Frameshift
		Variant at the cDNA level (NM_206538.4)	c.543dup	c.66delC	c.66delC	c.259C>T	c.259C>T	c.259C>T	c.289C>T	c.287del	c.188‐2A>C	c.188‐2A>C	c.679‐1G>A	c.287delG
		Variant at the protein level	p.Asn182GlnfsTer16	p.Ser23ValfsTer82	p.Ser23ValfsTer82	p.Gln87Ter	p.Gln87Ter	p.Gln87Ter	p.Arg97Ter	p.Gly96AlafsTer9	−	−	−	p.Gly96AlafsTer9
Variant characterisitcs		Methods	Makrythanasis et al.^12^	Yaron et al.^13^		Ucuncu et al.^14^			Makrythanasis et al.^12^	Basel‐Salmon et al.^15^	Makrythanasis et al.^12^			
		Maximum allele frequency in variant databases^1^	<0.00001	<0.000001	<0.000001	<0.00001	<0.00001	<0.00001	<0.00001	<0.00001	<0.00001	<0.00001	<0.000001	<0.0001
		ACMG classification	Pathogenic (PVS1, PM2)	Pathogenic (PVS1, PM2)	Pathogenic (PVS1, PM2)	Pathogenic (PVS1, PM2, PP3)	Pathogenic (PVS1, PM2, PP3)	Pathogenic (PVS1, PM2, PP3)	Pathogenic (PVS1, PM2, PP3)	Pathogenic (PVS1, PM2)	Pathogenic (PVS1, PM2, PP3)	Pathogenic (PVS1, PM2, PP3)	Pathogenic (PVS1, PM2, PP3, PP5)	Pathogenic (PVS1, PM2)
Epidemiological data		Ethnic group	Azerbaijani	Bukharan Jewish	Bukharan Jewish	Egyptian	Egyptian	Egyptian	Persian	Bedouin	Egyptian	Egyptian	Saudi	Saudi, Arab, Bedouin
		Gender/current age	M/8 y 10 m	M/14.5 y	M/26 y	F/8 y	F/8 y	F/6 y	F/11.3 y	F/5 y	M/7 y 5 m	F/2 y 11 m	F, M/11, 14 y	F‐ 5, M‐8
		Age at last examination	8 y 10 m	14.5 y	26 y	7 y	7 y	9 y	11 y	4 y 7 m	7 y 5 m	2 y 11 m	14 and 11 y	3 m–27 y
		Consanguinity/family history	+/−	+/+	+/+	+/+	+/+	+/+	+/+	+/−	+/+	+/+	+/+	+/+
Growth	At birth	Premature birth	−	+	+	+	+	−	−	−	−	−	−	NA
		Congenital microcephaly	NA	NA	NA	−2 SD	−2 SD	−1 SD	−	NA	−	−	−	−
		Low birth weight	−	+	+	−	−	−	−	−	−	−	−	−
	At last exam‐*n*	Low weight	−2.68 SD	Yes	−1.34 SD	−1 SD	−1 SD	−1 SD	−	+	−	−	−	−
		Microcephaly (congenital or acquired)	NA	+	−	Congenital	Congenital	−	−	−	Acquired	Acquired	−	−
Development		Age at first symptoms	Infancy (3–4 m)	Infancy	Birth, infancy	2.5 y	2.5	3 y	6 m	Infancy	3–4 m	3–4 m	3 and 2 y	NA
		Type of progression (rapid, moderate, slow)	NP	NP	NP	Moderate	Moderate	Moderate	Slow	NP	NP	NP	NP	NP
		DD/ID (severity)	Mild‐to‐moderate	Mild‐to‐moderate	Mild‐to‐moderate	Moderate‐to‐severe	Moderate‐to‐severe	Moderate‐to‐severe	Mild‐to‐moderate	Mild‐to‐moderate	Mild‐to‐moderate	Mild‐to‐moderate	+/2 mild‐to‐moderate	+12/13 mild‐to‐moderate^2^
		Failure to thrive	+	−	+	−	−	−	−	+	−	−	−	+4/13
		Regression in development	−	−	−	+	+	+	−	−	−	−	−	−
		Non‐ambulatory	−	−	−	+	+	+	−	−	−	−	−	−
Neurological symptoms		Dysarthria	+	−	−	+	+	+	+	−	+	+	−	−
		Poor speech	+	+	−	+	+	+	+	+	−	−	−	−
		Axial hypotonia	−	−	+	+	+	+	−	−	−	−	−	+3/5^2^
		Peripheral hypotonia	+	−	+	−	−	−	−	+	+	+	−	−
		Poor head control	−	−	−	+	+	+	+	−	−	−	−	−
		Truncal ataxia	NA	−	−	+	+	+	−	−	−	−	−	−
		Hyperkinetic/hypokinetic movement disorders	+	−	−	+	+	+	−	−	−	−	+	−
		Ataxic/clumsy gait	−	−	−	+	+	+	+	−	−	−	−	−
		Seizures, age of onset, type	−	−	−	+, 3 y.o., CP	+3, y.o., CP	+, 3 y.o., CP	+, 2 y.o., ST	−	−	−	+1, 3.5 y.o., FS	+6/13, infantile, MF, GTCS
Other systems and investigations		Facial dysmorphism	+	+	+	+	+	+	+	+	+	+	+	+
		Arachnodactyly	−	+	+	+	+	+	−	+	+	+	−	+3/13
		Elevated PTH	NA	+	+	NA	NA	NA	NA	NA	NA	NA	NA	+3/13^2^
		Other features	UIH, CHD	NC	MHN	−	−	VSD	−	−	CD	CD	−	+4/11 NC, +2/11 HN, +2/13 VSD, +5/13 UIH
		Brain MRI findings	A Short and thin CC	Abnormal HP and IBA	Abnormal HP and IBA	Thin CC, VMA	Thin CC, VMA	Thin CC, VMA	Normal	FMC	Normal	NA	Normal	Thin CC 3/13, Chiari I, 4/13, GMH 1/13 AM 3/13

S, subject; M, male; F, female; y, years; m, months; SD, standard deviation; NA, not available; NP, nonprogressive; CP, complex partial; ST, subclinical tonic; FS, febrile seizure; MF, multifocal seizures; GTCS, generalized tonic–clonic seizures; NC, nephrocalcinosis; MHN, mild hydronephrosis or hydroureter; CHD, congenital heart defect; PTH, parathyroid hormone; UIH, umbilical or inguinal hernias; CD, bilateral 5th digit clinodactyly; CC, corpus callosum; HP, hippocampi; IBA, initial brain atrophy; VMA, vermian atrophy; FMC, focal area of malformed cortex (thick and dysplastic) in the right frontal lobe; GMH, gray matter heterotopia; AM, abnormal myelination.

^1^ Database checked include GnomAD v3, gnomAD v2.1.1, TopMED Bravo, UKBiobank, Iranome, GME Variome, In‐house Database. The total number of alleles considered was ~1,314,000.

^2^ Follow‐up details.

## Results

### Clinical delineation

**Figure 1:**
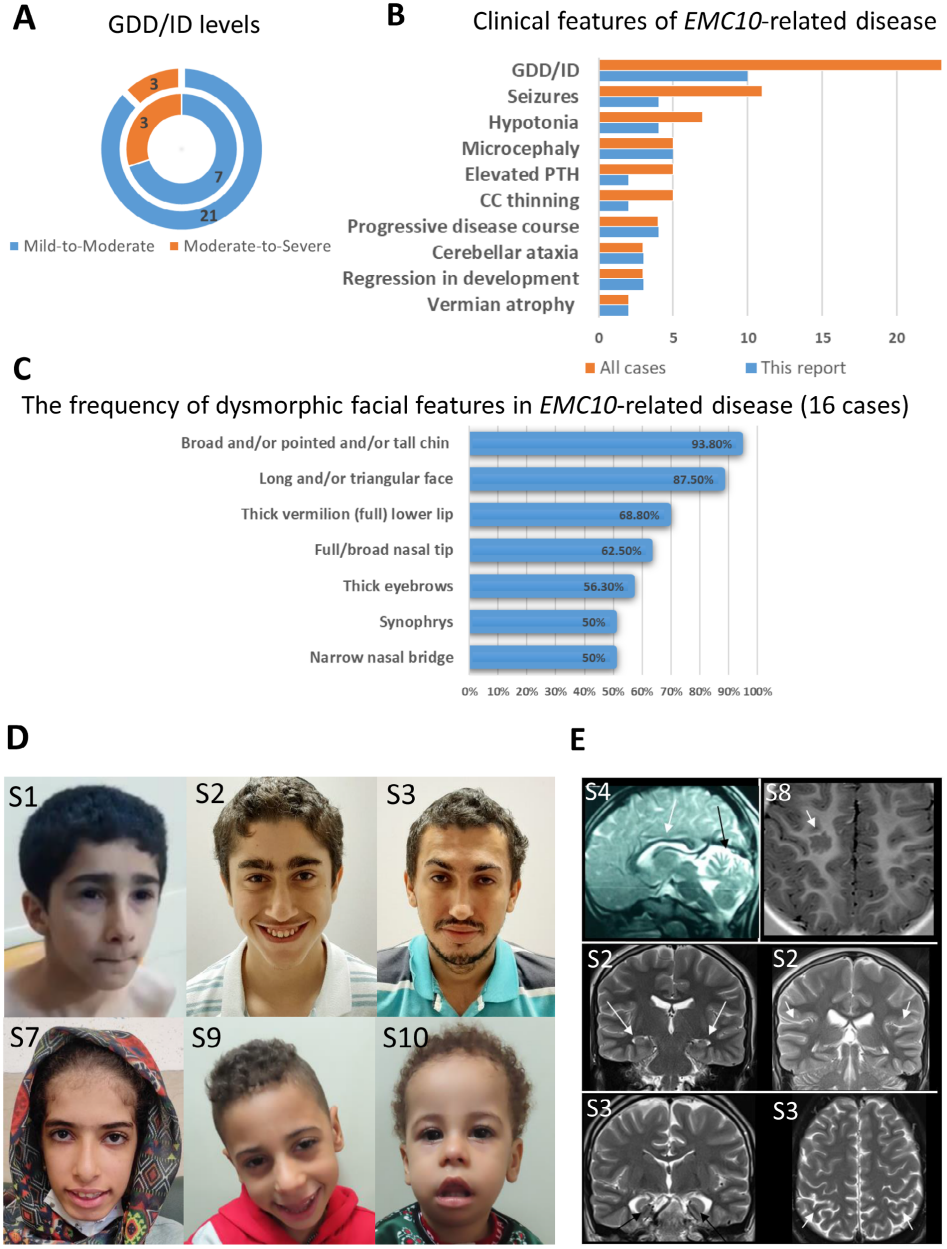
Clinical features of the cases with *EMC10*‐related NDD. (A) Levels of Global developmental delay/intellectual disability (GDD/ID): inner circle for cases reported here. Outer circles for all reported cases with biallelic *EMC10* variants. (B) Clinical features of *EMC10‐*related NDD: for cases reported here and for all reported cases with biallelic *EMC10* variants. PTH, parathyroid hormone; CC, corpus callosum. (C) The frequency of dysmorphic facial features in *EMC10*‐related disease (16 cases). This describes cases reported in the present study and all previously reported cases with available facial photos. (D) Facial photos of the affected individuals from this study: S1 – triangular face, thick eyebrows, narrow nasal bridge, pointed chin. S2 – long face, curly hair, low anterior hairline, right anterior cowlick, widow's peak, thick eyebrows (caterpillar‐like), synophrys, up‐slanting palpebral fissures, hypotelorism, lateral infraorbital creases, narrow nasal bridge, short philtrum, full nasal tip, tall and broad chin. S3 – Curly hair, prominent supraorbital ridges, thick and highly‐arched eyebrows, synophrys, up‐slanting right palpebral fissure, narrow nasal bridge, full nasal tip, short philtrum, full lips, and pointed chin. S7 – Long face, tall forehead, bifrontal narrowing, low columella, high anterior hairline, thick eyebrows, synophrys, up‐slanting palpebral fissures, narrow nasal bridge, full nasal tip, short philtrum, wide mouth, full lower lip, and broad chin. S9 – long face, curly hair, tall forehead, high anterior hairline, full cheeks, full nasal tip, low‐hanging columella, wide mouth, full lower lip, and broad chin. S10 ‐ long face, sparse and curly hair, high anterior hairline, tall forehead, bifrontal narrowing, full cheeks, large ear lobes, full nasal tip, tented upper lip, full and everted lower lip, midline depression over lower lip, broad chin, retrognathia. (E) Brain MRI scans. In the upper left row CC thinning (white arrows) and vermian atrophy (black arrow) in S4. On the right, dysplastic malformed cortex (arrowhead) is shown in S8. The middle and lower rows show mild brain atrophy and hippocampal dysmorphisms in two subjects from family 2 (S2 and S3). In the middle row white arrows point at small hippocampi while in the last row, black arrows show rounded and simplified hippocampal structure. In these affected individuals, mild cortical atrophy is demonstrated by the enlargement of cortical sulci (white arrowheads in the right middle and bottom rows). NDD, neurodevelopmental disorder.

The main phenotypic features of the present cohort are summarized in (Fig. 1); Table 1 and Table S1. Detailed clinical history is provided in the Supplementary Case Reports.

The cohort is composed of 10 affected individuals including six females and four males, all of whom are currently alive with a mean age of 9.8 ± 6.5 years (range 5–26). All cohort members were born to consanguineous unions. Four cases were born prematurely with confirmed congenital microcephaly (2/10), low birth weight (2/10), and obstructive hydrocephalus (1/10). Failure to thrive was reported in 3/10 (30%) affected individuals. The disease was of infantile‐onset (≤12 months) in 7/10 and manifested at 2.5 and 3 years old in three siblings from family 3. GDD ranging from mild to severe was a common manifesting feature in the cohort. Concerning developmental domains, language acquisition skills were significantly delayed in all cohort members, three siblings from family 3 failed to acquire independent ambulation by the ages 6 and 7 years old, and S1 had difficulties with fine motor skills. The disease course was non‐progressive in 6/10 (60%) cases, and in others, the progression had either slow (family 4) or moderate (family 3) rates. Three siblings from family 3 had slow regression in motor skills.

Upon the most recent follow‐up at a mean age of 9.5 ± 6.2 years, 5/10 (50%) cases had microcephaly and 3/10 (20%) cases had small weights. Congenital heart disease was found in 2/10 (20%) cases. GDD/ID ranging from mild to severe was present in all cohort members. Most of the cases (9/10, 90%) had limited language abilities. Mild axial (4/10, 40%) and peripheral (5/10, 50%) hypotonia, truncal ataxia (3/10, 30%), dysarthria (7/10, 70%) with ataxic or clumsy gait (4/10, 40%), and hyperkinetic movement disorders (4/10, 40%) were frequent neurological findings. Complex partial seizures controlled by sodium valproate were reported in siblings from family 3. Their electroencephalography showed bilateral frontotemporal epileptogenic dysfunction. The proband from family 4 had subclinical tonic seizures from the age of 2 years responsive to carbamazepine.

Facial photographs were reviewed for six affected individuals from four families. This included five children and one adult (Fig. 1D). Published photographs of 10 previously reported patients were also reviewed.^8^, ^9^ These included eight children, one adolescent and one adult from five families. The dysmorphic features were described based on terminology recommended by Elements of Morphology. Where this classification had no descriptive term available for a dysmorphic feature seen in a patient, HPO terminology was used instead. Detailed facial dysmorphic features for all affected individuals whose photographs were reviewed are shown in Table S2 and dysmorphic features are tabulated using HPO codes in Table S3 to generate the frequency of each feature. Figure 1C shows the most frequently seen facial dysmorphic features of *EMC10‐*related NDD. Additionally, arachnodactily and bilateral fifth digit clinodactyly were present in 8/10 (80%) and 2/10 (20%) subjects, respectively.

Siblings from Family 2 had repeatedly (S2) or transiently (S3) elevated parathyroid hormone levels with nephrocalcinosis (S2), osteoporosis (S2), hyperuricemia (S3), and hydronephrosis (S3). Three cases from Shao et al. report^9^ were confirmed to have elevated parathyroid hormone levels. Plasma and urine metabolic tests available from the presently reported seven cases were unremarkable.

Full brain MRI exams were available in six cases, while in two subjects from family 3 only midline sagittal slices could be reviewed. The abnormal findings include a thin corpus callosum (CC) (2/8), abnormal hippocampi (small and/or rounded) and initial brain atrophy (2/6), vermian atrophy (2/6), and a focal cortex malformation (1/6) (Fig. 1C). Two subjects from family 4 and family 6 showed normal findings.

### Genetic findings

**Figure 2:**
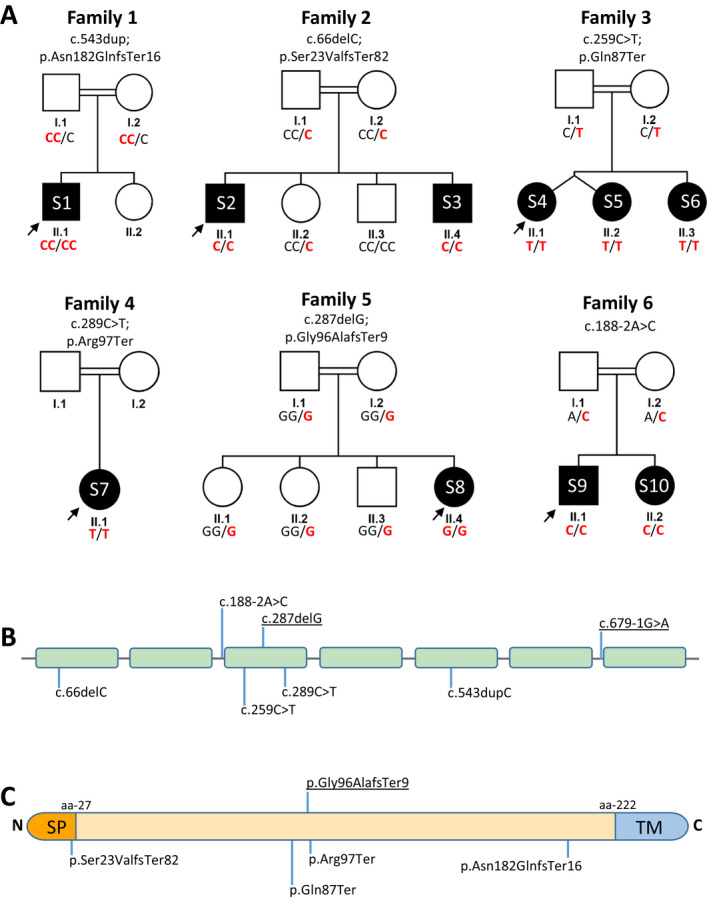
Pedigrees and genetic data. (A) Pedigrees of the six families described. The variant is indicated by the bold red letter. Square = male; circle = female; black filled symbol = affected individual; white symbols = unaffected individuals. Double lines indicate consanguinity. (B) Schematic representation of the seven exons in the EMC10 gene. Previously described variants are underlined. (C) Schematic diagram of the EMC10 protein. The orange section represents N terminal signal peptide (SP), the yellow section represents the topological domain, and the blue section shows the C terminal transmembrane domain (TM).^22^

We identified five new and one previously reported recurrent homozygous LOF *EMC10* (NM_206538.4) variants in six consanguineous families as follows: c.543dup; p.Asn182GlnfsTer16 in family 1, c.66delC; p.Ser23ValfsTer82 in family 2, c.259C>T; p.Gln87Ter in family 3, c.289C>T; p.Arg97Ter in family 4; c.287del; p.Gly96AlafsTer9 in family 5 and c.188‐2A>C in family 6 (Table 1). These variants reside within sizeable regions of homozygosity and are segregated with the disease in the families (Fig. 2A). The location of the variants did not indicate any cluster sites in the gene nor in the protein (Fig. 2B–C). Inspection of multiple public and private variant databases including gnomAD (v2.1.1 and v3.1.2), TopMED, Iranome, GME Variome, UK Biobank, and our in‐house database of ~25,000 exomes (total number of alleles ~1,314,000) revealed that the variants are absent or with extremely rare allele frequency reported ranging from <0.0001 to <0.000001. Moreover, according to ACMG classification, all reported variants were considered pathogenic (Table 1). No unaffected individuals reported homozygous for any of these variants. Upon further re‐analysis of the exomes, no additional clinically relevant variants were identified in any of the families.

## Discussion

The EMC has been thought to be implicated in several cellular processes though its primary function remains debatable.^16^ The EMC family was first identified in yeast as a multi‐protein transmembrane complex, where it was thought to be an ER‐mitochondria tether that interacts with the outer membrane protein Tom5 of the Translocase of the mitochondrial outer membrane complex (TOM).^16^, ^17^ Defective EMC has been suggested to decrease, but not abolish, the insertion and proper function of multiple transmembrane proteins.^9^ EMC has also been implicated in the folding of multipass membrane proteins.^18^ Several recent studies have shown that impaired ER‐mitochondria crosstalk could be involved in neurodegenerative diseases.^19^

*EMC10* was first identified as a disease‐associated gene in two independent studies utilizing ES in individuals with GDD.^8^, ^9^ The recurrent *EMC10* c.287delG, p.Gly96AlafsTer9 variant identified in seven families significantly reduced *EMC10* RNA expression and resulted in an unstable truncated EMC10 protein. Staining for EMC10 in postmortem human infant brain showed colocalization with neuronal markers MAP2 and NeuN, markers specifically expressed in neuronal perikarya and dendrites and the nucleus of post‐mitotic neurons, respectively.^9^, ^20^

Here, we report 10 affected individuals from six unrelated families with five previously unreported and one reported recurrent biallelic LOF *EMC10* variants. Replicating the features described in the previous *EMC10* cohort, the current cases exhibited uniform GDD/ID (with prominent delay in verbal milestones) and facial dysmorphism, and variably presented with failure to thrive, axial hypotonia, seizures, microcephaly, CC thinning, and cardiac and renal pathology. The dysmorphic facial feature most commonly shared between the previous and present *EMC10* cohorts was a long and/or triangular face. In contrast, expanding the clinical spectrum of the *EMC10*‐related NDD, the cases reported here presented with dysarthria, persistent peripheral hypotonia, movement disorders, gait ataxia, and vermian atrophy together with noticeable growth impairment, and hyperparathyroidism. Furthermore, the progressive disease course and regression in motor skills seen in the current cohort appear to expand the phenotype severity of the *EMC10*‐related NDD towards the more severe end. Being based on the cumulative phenotypic analysis of all available cases with defective *EMC10*, Figure 1B depicts the most common clinical features of *EMC10‐*related NDD.

Akin to defective *EMC1* presentation, we report signs of cerebellar ataxia with detectable loss of cerebellar volume, persistent hypotonia, movement disorders, CC thinning, and anomalies in the hippocampi in our cohort.^4^, ^5^, ^6^, ^7^ The presence of truncal ataxia in the *EMC10* cohort might replicate the abnormal gait observed in the homozygous *emc10*‐knockout mouse models characterized by the International Mouse Genotyping Consortium (https://www.mousephenotype.org/data/genes/MGI:1916933).^21^ The *EMC1* cohort has shown progressive brain atrophy, most pronounced in the cerebellum, suggesting a neurodegenerative process.^4^ The presence of motor regression, progressive disease course, and supratentorial brain atrophy in the current cohort might also imply underlying neurodegeneration. The described expansion of the defective *EMC10* phenotype suggests that clinical features might overlap between *EMC1* and *EMC10* defects.

Given that *EMC10* is a ubiquitously expressed gene and knockout mouse (*emc10*−/−) models showed decreased bone mineral content (https://www.mousephenotype.org/data/genes/MGI:1916933),^21^ the hyperparathyroidism together with osteoporosis and nephrocalcinosis observed in F2:S1, F2:S2, and the three previously reported cases might be linked to dysfunctional *EMC10* presentation.

Regarding the genotype–phenotype correlation, cases with stop gained variants had more severe symptoms including regression combined with persisting axial hypotonia, seizures, and ataxic gait.

In conclusion, this report expands the genetic and clinical spectrum of *EMC10* deficiency, provides a comprehensive dysmorphological assessment, and highlights an overlap between the clinical features of *EMC10‐* and *EMC1‐*related NDD.

## Conflict of Interest

The authors have no conflict of interest to report.

## Web Resources

gnomAD, https://gnomad.broadinstitute.org/.

Ensemble, https://www.ensembl.org/index.html.

Uniprot, https://www.uniprot.org/.

GeneMatcher, https://genematcher.org/.

OMIM, https://www.omim.org/.

Iranome, http://www.iranome.ir/.

Varnomen, http://varnomen.hgvs.org/.

Varsome, https://varsome.com/.

Database of Genome Variants, http://dgv.tcag.ca/.

UKBB, https://www.ukbiobank.ac.uk/.

GME Variome, http://igm.ucsd.edu/gme/data‐browser.php.

TOPMed, https://bravo.sph.umich.edu/freeze8/hg38/gene/snv/EMC10.

## Supporting information


**Data S1.** Supplementary case reports.

**Table S1.** Extended clinical table.

**Table S2.** Detailed facial dysmorphic features for all subjects whose photographs were reviewed.

**Table S3.** Frequency of each dysmorphic feature in *EMC10* cohort with available facial photos.
