# Supplementary material for: Biallelic loss of EMC10 leads to mild to severe intellectual disability
Source: Ann Clin Transl Neurol. 2022 Jun 9;9(7):1080–9. doi: 10.1002/acn3.51602 (PMC9268894; doi:10.1002/acn3.51602)
Supplement: Supplementary file 1 — Data S1. Supplementary case reports. [file ACN3-9-1080-s002.docx]

SUPPLEMENTARY CASE REPORTS

**Family 1**

The index case (S1) is a 9-year-old boy born full-term to consanguineous parents of Azerbaijani origin. His birth weight was at the 10th percentile, and due to persistent failure to thrive his parent sought medical attention when he reached the age of 3-4 months. Later he presented with mild global developmental delay (GDD). He was able to sit independently at 9 months old and acquired independent walking at 1 year old. From the age of 16 months, he started using first words.

Upon his follow-up, at 8 years and 10 months old he was significantly underweight (below the 5th percentile) with a head circumference (the 10th percentile). He presented with intellectual disability and inadequately acquired language skills. Interaction with family members was preserved. Neurological examination revealed mild peripheral hypotonia, difficulties in fine motor skills, and choreoathetoid movements in the limbs. He did have a history of seizures. Apart from inguinal and testicular hernia, the examination and investigation of his muscular-skeletal system and internal organs were unremarkable. He had dysmorphic facial features including a triangular face, thick eyebrows, narrow nasal bridge, and pointed chin. Overall, his disease had a non-progressive course. Brain magnetic resonance imaging (MRI) showed a short and thin corpus callosum.

**Family 2**

This family presents with 2 affected male siblings (proband S2 and affected sibling S3) aged 14.5 and 26 years old respectively at the last follow-up. Both of them were born prematurely to consanguineous parents of Bukharan Jewish origin. Their birth weights were below the 3rd percentile, and both of them had abnormal neonatal findings including unilaterally undescended testis in S2 and obstructive hydrocephalus with aqueduct stenosis in S3 found on the computed tomography of the brain on the 3rd day of life. They manifested with global developmental delay, which was mild in S2 with predominant delay in language acquisition and associated with hypotonia, feeding difficulties, and failure to thrive in S3. At the age of 3 days, S3 had ventriculoperitoneal shunting (VPS) due to patent narrow aqueduct and obstructive hydrocephalus. Overall, the disease course in both siblings was non-progressive. Upon the most recent physical examination both siblings had arachnodactyly and facial dysmorphism encompassing long face, curly hair, low anterior hairline, right anterior cowlick, widow's peak, thick eyebrows (caterpillar-like), synophrys, up-slanting palpebral fissures, hypotelorism, lateral infraorbital creases, narrow nasal bridge, short philtrum, full nasal tip, tall and broad chin in S2 and curly hair, prominent supraorbital ridges, thick and highly-arched eyebrows, synophrys, up-slanting right palpebral fissure, narrow nasal bridge, full nasal tip, short philtrum, full lips, pointed chin in S3. Additionally, S2 had microcephaly (-2.5SD) and undescended atrophic (vanishing) right testis. Neurological examination revealed moderate ID with inadequate language skills in S2 and mild ID in S3. There was very mild axial and peripheral hypotonia in S3, and both siblings had no history of seizures.

Investigations showed elevated parathyroid hormone (PTH) (124 pg/ml) with nephrocalcinosis in S2 and parathyroid adenoma was ruled out. Transient mildly elevated PTH and borderline hypercalciuria with the spontaneous resolution was reported in S3. Echocardiography was normal in both siblings, while renal ultrasound revealed bilateral hyperechogenic kidneys and cortical thinning in S2 and left hydronephrosis in S3. Genetic testing, including FMR1 expansion, karyotype, myotonic dystrophy, ARX sequencing, maternal X inactivation, was negative in S2.

Brain MRI investigations done in S2 at 11 months of age showed abnormal hippocampi and initial brain atrophy and MRI in S3 at age 25-year-old similarly showed VPS in situ with abnormal hippocampi and initial brain atrophy.

**Family 3**

This family has 3 affected female siblings (S4 and S5 are twins and S6 is a younger sibling) born to consanguineous Egyptian parents. The twin siblings were born prematurely and are currently aged 8 years old, while the younger sibling (S6) is a product of unremarkable full-term pregnancy and is currently aged 6 years old. Congenital heart disease was present in S6. All siblings presented with severe GDD and congenital microcephaly followed by slow motor regression. They sat independently at 2 years old, started using their first words at 3 years old, and failed to acquire an independent gait. On the most recent follow-up at ages 7 (S4 and S5) and 6 (S6) years old, all siblings had arachnodactyly and facial dysmorphism including long triangular face, dolichocephaly, prominent nasal root, long philtrum, thin upper lip, midfacial hypoplasia, low set ears, and short neck. Additionally, both twins had microcephaly. Neurological examination showed intellectual disability, axial hypotonia with truncal ataxia, and hyperkinetic movements in the limbs in all affected siblings. They all had dysarthric speech with gait ataxia on walking with support. All siblings had complex partial seizures that started from age 3 years and recurring once a week on sodium valproate. Electroencephalography showed bilateral frontotemporal epileptogenic dysfunction. Investigations including plasma and urine organic and amino acids, electromyography, and renal ultrasound were unremarkable. Brain MRI in all affected siblings uniformly showed severe CC thinning and vermian atrophy.

**Family 4**

The index case (S7) is an 11-year-old female born full-term after unremarkable pregnancy to consanguineous parents of Persian ethnicity. Her neonatal period was unremarkable. She manifested with delayed head control at 6 months old and later was found to have GDD. She sat at 11 months old, walked independently at 16 months old, and started using her first words at 2.5 years old. The disease had slow rates of progression and upon her most recent examination at 11 years old she exhibited ID with limited verbal communication. She had dysarthria and a clumsy gait. There was a history of subclinical tonic seizures from the age of 2 years controlled with Carbamazepine. There were dysmorphic facial features including a long face, tall forehead, bifrontal narrowing, low columella, high anterior hairline, thick eyebrows, synophrys, up-slanting palpebral fissures, narrow nasal bridge, full nasal tip, short philtrum, wide mouth, full lower lip, broad chin. She had neither musculoskeletal nor internal organ abnormalities. Metabolic testing in blood and urine was unremarkable and brain MRI was reported as normal.

**Family 5**

The proband (S8) is a 5-year-old girl born full-term after unremarkable pregnancy to consanguineous and healthy parents of Bedouin ethnicity. She manifested with mild-to-moderate global developmental delay and failure to thrive. She walked from the age of 2 years and 2 months and used her first words from age 1 year and 11 months. At the most recent follow-up at 4 years 7 months she was underweight with height and head circumference below the 10^th^ and 14^th^ percentiles respectively. She did not have microcephaly. Dysmorphic features included arachnodactyly, triangular face with wide forehead, mild frontal bossing, prominent metopic ridge, and hypertelorism, combined with epicanthal folds, cupped protruding ears, mild macrostomia with protruding tongue, widely spaced teeth, and thickened gums. She had intellectual disability, peripheral hypotonia, and poor speech. MRI at age 1 year and 3 months showed a focal area of the malformed cortex (thick and dysplastic) in the right frontal lobe.

**Family 6**

The index case is a 7.5-year-old male (S9) born after uneventful pregnancy and birth to consanguineous parents of Egyptian origin. At age 3-4 months old, he manifested global developmental delay leading to late unassisted sitting (at 1-year-old), independent walking (at 2.5 years old), and language acquisition (the first word at 3 years). Since then, his motor delay has been mild but he had a more pronounced cognitive delay. He also had repetitive movements and laughing and mild autistic features. His disease was not progressive; conversely, slow improvement has been noticed. Poor speech acquisition and intellectual disability were reported as the main medical problems. On his most recent physical examination at age 7.5 years, he was microcephalic (-3SD) with an IQ score of 50 and poor language (only a few words). He had preserved ocular contact and could interact with others, but communication was limited due to impaired cognition, hyperactivity, and mild autistic features. Neurological examination revealed dysarthria and mild peripheral hypotonia. His face was dysmorphic encompassing a long face, curly hair, tall forehead, high anterior hairline, full cheeks, full nasal tip, low-hanging columella, wide mouth, full lower lip, broad chin. His limbs showed arachnodactyly and bilateral 5th digit clinodactyly. Metabolic tests, ultrasound investigations, and neurophysiological studies were unremarkable. His creatine phosphokinase level was 197 mcg/L. Brain MRI at 4 years old showed thin short corpus callosum and underdeveloped hippocampal.

His affected sister (S10), currently aged 2.9 years was the product of unremarkable pregnancy and delivery. Global developmental delay had been noticed from age 3-4 months with late independent sitting (at 1 year 2 months) independent (at 2 years and 4 months), and use of the first words at 2y 6m (only 2 words). She had the following dysmorphic features: a long face, sparse and curly hair, high anterior hairline, tall forehead, bifrontal narrowing, full cheeks, large ear lobes, full nasal tip, tented upper lip, full and everted lower lip, midline depression over the lower lip, broad chin, retrognathia, and fifth finger clinodactyly. Her cognitive, behavioural, and neurological symptoms together with investigation results were similar to her affected brother.
